# Effects of Serum Cytochrome c on Contrast-Induced Nephropathy in Patients with ST-Elevation Myocardial Infarction Undergoing Percutaneous Coronary Intervention

**DOI:** 10.1155/2019/9357203

**Published:** 2019-01-23

**Authors:** Chengchun Tang, Jiantong Hou, Gaoliang Yan, Yong Qiao, Dong Wang, Boqian Zhu, Bo Liu, Erfei Luo, Abdul Qadir Nawabi, Long Chen

**Affiliations:** Department of Cardiology, Zhongda Hospital of Southeast University Medical School, Nanjing 210009, China

## Abstract

**Background and Aims:**

Contrast-induced nephropathy (CIN) is a relatively infrequent complication after percutaneous coronary intervention (PCI) in patients with ST-elevation myocardial infarction (STEMI). However, little is known about the association between cytochrome c (cyt c) and increased risk of CIN. We conducted this study to explore the impact of serum cyt c on the occurrence of CIN.

**Methods:**

We prospectively examined cyt c levels before undergoing PCI in 240 patients with STEMI. The logistic regression was performed to identify the independent risk factors for the occurrence of CIN. The receiver operating characteristic (ROC) analysis was employed to evaluate the predictive value of cyt c for the occurrence of CIN.

**Results:**

29 patients (12.1%) had developed CIN after the PCI procedure. The cyt c levels at baseline were significantly higher in patients who developed CIN than those in non-CIN group (0.65±0.08 versus 0.58±0.1; P = 0.001). The multivariate logistic regression showed that cyt c was an independent risk factor for the occurrence of CIN (OR, 7.421; 95% CI, 6.471–20.741; P = 0.034) after adjusting for age, history of hypertension and diabetes mellitus, levels of creatinine, uric acid, and glucose. The ROC curve analysis showed that the area under the curve of cyt c was 0.697 (95% CI, 0.611–0.783; P = 0.001), and cyt c > 0.605 ng/mL predicted CIN with sensitivity of 79.3% and specificity of 56.9%.

**Conclusion:**

Our results show that a higher cyt c level was significantly associated with the occurrence of CIN after PCI in STEMI patients. This study has been registered in the Chinese Clinical Trial Registry. The clinical trial registration number is ChiCTR1800019368.

## 1. Introduction

Contrast-induced nephropathy (CIN) is a relatively common and serious complication after intravascular injection of iodinated radiographic contrast medium in patients with ST-elevation myocardial infarction (STEMI).The occurrence of CIN leads to not only prolonged hospitalization of patients but also an increase in mortality after percutaneous coronary intervention (PCI), which seriously affects the clinical prognosis of coronary heart disease [1]. CIN accounts for 11% of the causes of hospital-acquired acute kidney injury (AKI) and is the third of all causes [2]. CIN is defined as an increase in serum creatinine of more than 25% or 44.2 mmol/L^−1^ 48 to 72 hours after contrast medium administration without evidence of other causes [3]. However, serum creatinine has a certain hysteresis in the diagnosis of CIN. Therefore, it is particularly important to find more rapid, reliable, and sensitive indicators for early diagnosis and screening of CIN in high-risk patients.

The pathogenesis of CIN has not been completely explained by a single mechanism, but apoptosis plays an key role in the development of CIN [1, 4–6]. Cytochrome c (cyt c) is a 14 kDa hemoprotein that normally attaches to the outer surface of the inner mitochondrial membrane in a form that binds to cardiolipin and plays an important physiological role in oxidative phosphorylation by transferring electrons between complex III and complex IV [7, 8]. The cyt c is released from the mitochondrial membrane to the cytoplasm, which activates caspase 3 and eventually leads to apoptosis [9, 10]. There were studies shown that elevated levels of circulating cyt c were closely associated with myocardial infarction, acute kidney injury, and systemic inflammatory response syndrome [11–13]. Recently, several novel renal biomarkers have been proposed to predict early CIN, including Antithrombin III, neutrophil gelatinase-associated lipocalin (NGAL), cyt c, and kidney injury marker 1 (KIM-1) [14, 15]. However, no relevant report is currently available regarding whether cyt c is associated with CIN after PCI in STEMI patients. In this study, we aimed to investigate whether preoperative cyt c can be used as an early diagnostic marker for CIN after PCI in patients with STEMI.

## 2. Materials and Methods

### 2.1. Study Subjects

The study was approved by the medical ethics committee of the Zhongda Hospital of Southeast University Medical School, and all patients obtained informed consent. In this prospective observational study, 240 adult patients with STEMI between March 2017 and February 2018 who agreed to receive primary PCI treatment in the Cardiology Department of Zhongda Hospital Southeast University were enrolled. The enrollment criteria were the definite diagnoses of STEMI. The exclusion criteria were as follows: (1) end-stage renal disease requiring dialysis; (2) allergy to contrast agent; (3) intravascular administration of contrast medium within 2 weeks; (4) autoimmune diseases; (5) malignant tumor; (6) infectious diseases; and (7) cardiac shock.

### 2.2. Study Protocol and Definition

CIN is defined as an increase in serum creatinine of more than 25% or 44.2 mmol/L^−1^ 48 to 72 hours after contrast medium administration without evidence of other causes. The diagnosis of STEMI relies on the typical symptoms of angina pectoris, changes in electrocardiogram, and the measurements of myocardial necrosis markers. The patients with STEMI were divided into CIN and non-CIN groups according to the presence or absence of CIN, respectively. Baseline cyt c and serum creatinine levels were tested before angiography. Troponin I, low-density lipoprotein cholesterol (LDL-C), uric acid, urea nitrogen, glucose, *γ*-glutamyl transpeptidase (*γ*-GT), albumin, and hemoglobin were also measured. The cyt c was measured using a commercially available enzyme-linked immunosorbent assay (Quantikine; R&D System Inc, Minneapolis, MI). Troponin I, serum creatinine, LDL-C, uric acid, urea nitrogen, glucose, *γ*-GT, albumin, and hemoglobin were all tested on AU5800 autobiochemistry analyzer (Beckman Coulter, USA). Regular serum creatinine test during 48 to 72 hours following PCI was performed to diagnose CIN. The estimated glomerular filtration rate (eGFR) was calculated using the Modification of Diet in Renal Disease (MDRD) formula evaluating patients' renal function.

### 2.3. Coronary Interventions and Medications

All patients were operated on by cardiologists specialized in intervention treatment. The corresponding diseased vessels were treated according to the specific results of coronary angiography. The contrast medium applied was iodixanol (GE healthcare, UK). This agent is nonionic with isoosmolarity. Hydration therapy was given depending on the eGFR level; patients with eGFR< 60mL/min/1.73m^2^ were given hydration. During hydration, isotonic saline solution was intravenously given at 1.0–2.0 mL/kg/h for 3–12h preoperatively and 24 h preoperatively. After PCI, the rational use of medications such as aspirin, ticagrelor, *β*-receptor blockers, ACEI or ARB, CCB, statin, and diuretics was left to clinicians according to clinical conditions.

### 2.4. Statistical Analysis

Data were analyzed using the SPSS 17.0 (SPSS Inc., Chicago IL, USA). The measured data were presented as mean ± standard deviation or median and interquartile range. The Kolmogorov-Smirnov (K-S) test was applied to test normal distribution. The Student's *t*-test was applied to test the differences between continuous variables groups. Nonparametric Mann–Whitney *U* test was conducted to test the differences between nonnormal distribution groups. The categorical variables were tested using Chi-square test. Logistic regression model was performed to identify the influencing factors associated with CIN. The receiver operating characteristic (ROC) analysis was employed to evaluate the predictive value of cyt c for CIN and determine the best cutoff value of cyt c. P values < 0.05 were considered statistically significant.

## 3. Results

### 3.1. Baseline Clinical Characteristics

The study population consisted of 240 STEMI patients. The mean age was 64.03 ± 13.07 years, and 179 (74.6%) were males. Overall, 29 patients (12.1%) developed CIN after PCI procedure. Patients in the CIN group had a significantly older age (71.07±13.77 years versus 63.06±12.7 years; *P* = 0.002) and significantly lower LVEF (*P* = 0.02) compared with patients in the non-CIN group. Statistically significant differences were also noted in history of hypertension or diabetes mellitus, left ventricle inner diameter, hydration, the use of ACEI/ARB and diuretics, and hospitalization days (*P* < 0.05) between the CIN group and the non-CIN group. There were no significant differences in gender, smoking, systolic blood pressure, diastolic blood pressure, heart rate, contrast dose, number of diseased vessels, and hospitalization costs between the CIN group and the non-CIN group (*P* > 0.05) (Table 1).

### 3.2. Baseline Laboratory Data

The cyt c levels at baseline were significantly higher in patients who developed CIN than those who did not (0.65±0.08 versus 0.58±0.1;P = 0.001). In addition to having elevated cyt c, patients in the CIN group had higher baseline creatinine, urea nitrogen, and glucose and significantly lower baseline albumin and hemoglobin levels than those in the non-CIN group (P < 0.05). (Table 2).

### 3.3. Logistic Regression Analysis of Risk Factors on Predicting CIN

Logistic regression analysis was performed to identify independent risk factors for the occurrence of CIN. After adjusting for age, history of hypertension and diabetes mellitus, levels of creatinine, uric acid, and glucose, cyt c was significantly associated with an increased risk of CIN (OR, 7.421; 95% CI, 6.471–20.741; *P* = 0.034). Additionally, diabetes mellitus (OR, 1.625; 95% CI, 1.121–4.64; *P* = 0.031) and levels of serum creatinine (OR, 1.025; 95% CI, 1.002–2.436; *P* = 0.032) were also independent risk factors of CIN (Table 3).

### 3.4. Sensitivity and Specificity of cyt c on Predicting CIN

**Figure 1:**
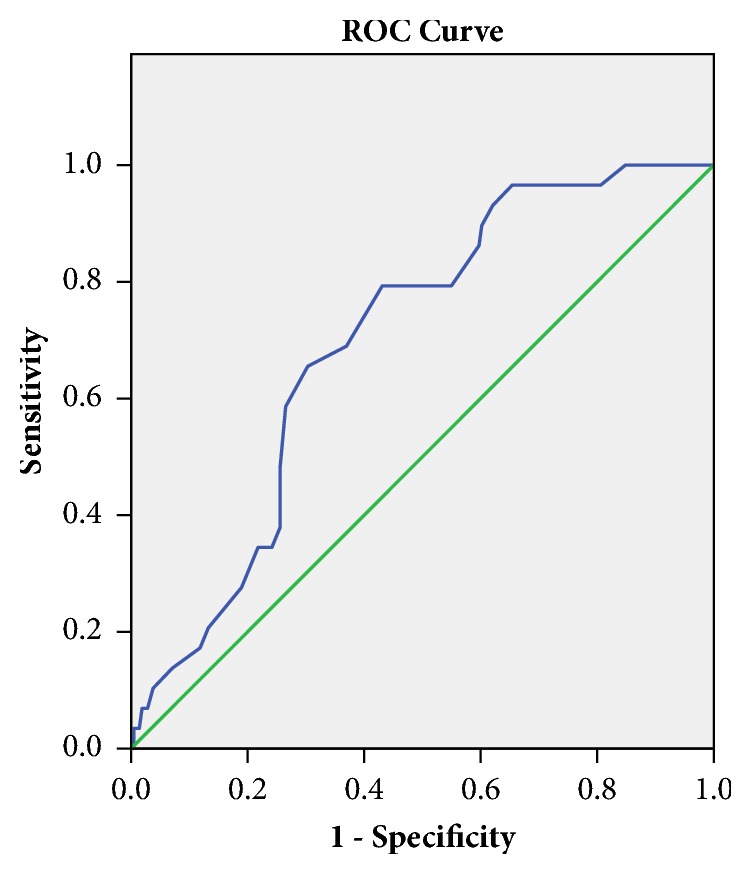
Schematic of the ROC curve for CIN prediction by cyt c. The area under the ROC curve for predicting CIN of cyt c was 0.697 with sensitivity of 79.3% and specificity of 56.9%.

The ROC curve analysis showed that the area under the curve of cyt c was 0.697 (95% CI, 0.611–0.783; *P* = 0.001). The optimum cutoff point of cyt c was 0.605, with sensitivity of 79.3% and specificity of 56.9% (Figure 1).

## 4. Discussion

In this study, we evaluated the impact of circulating cyt c levels on the risk of CIN in patients with STEMI undergoing PCI. Our data suggested that elevated levels of circulating cyt c are significantly associated with an increased risk of CIN. Elevated circulating cyt c levels are an independent risk factor for occurrence of CIN.

CIN is a common complication that occurs after the use of iodinated contrast agents. CIN is more likely to occur in unplanned coronary interventions. Although the criminal blood vessels were successfully opened in the early stage, CIN is still one of the short-term and long-term adverse prognostic risk factors after PCI in patients with acute coronary syndrome (ACS)[16]. Many risk factors can contribute to the development of CIN, such as reduced intravascular volume, advanced age, diabetes mellitus, chronic renal insufficiency, and extensive use of contrast agents [17]. Although great efforts have been made in the prevention and treatment of CIN, the results are still unsatisfactory and inconsistent [18, 19]. Therefore, it is crucial to search for an early sensitive indicator to predict the occurrence of CIN.

So far, little is known about the pathogenesis of CIN. Its pathogenesis may include acute renal ischemia, endothelial dysfunction, oxidative stress, and apoptosis [1, 4–6, 20]. Previous studies showed that contrast agents induce renal tubular cell apoptosis through ROS pathway, stress kinase pathway, and intrinsic apoptotic pathway [21]. The intrinsic pathway is activated by various intracellular and extracellular stresses, and its signal is mainly concentrated in mitochondria [22, 23]. The cyt c is a key factor in initiating mitochondrion-mediated apoptosis pathway and is closely related to cell apoptosis. The cyt c is located in the surface of inner mitochondrial membrane and plays a key role in mitochondrial energy metabolism. It can be released from mitochondria to cytosol when cells undergo apoptosis under various factors. There were studies which had shown that circulating cyt c levels are associated with electrocardiographic and angiographic signs of impaired myocardial reperfusion, extension of infarct size, and 1-year mortality [24, 25]. Moreover, cyt c is an important marker of the occurrence and development of acute kidney injury [11]. In this study, we found that circulating cyt c levels at baseline were significantly higher in CIN group than those in non-CIN group. We speculated that elevated cyt c levels may induce CIN by causing mitochondrial apoptosis. Further studies are needed to be conducted to prove this hypothesis.

Mitochondria are a network of plastic organelles that regulate the level of reactive oxygen species (ROS), apoptosis, and cytosolic calcium [26]. Mitochondrial dysfunction causes a decrease in ATP production, alterations in cellular structure and functions, and the reduction of renal function. ROS can trigger cell apoptosis by causing the release of cytochrome c, leading to mitochondrial dysfunction [27]. There was a study that showed that contrast administration induced significant increase in cyt c of the cytosol [28]. This indicated that cyt c might be a potential biomarker for the assessment of risk of CIN, as well as serving as a novel therapeutic target.

In this study, univariate logistic regression analysis was performed to screen for risk factors of CIN. The multivariate logistic regression was performed to further screen for independent risk factors of CIN. The multivariate logistic regression showed that cyt c was an independent risk factor for the occurrence of CIN (OR, 7.421; 95% CI, 6.471–20.741; P = 0.034) after adjusting for age, history of hypertension and diabetes mellitus, levels of creatinine, uric acid, and glucose. The ROC curve analysis showed that the area under the curve of cyt c was 0.697 (95% CI, 0.611–0.783; *P* = 0.001). When cyt c > 0.605 ng/mL, its predicted value of CIN has a sensitivity of 79.3% and a specificity of 56.9%. This indicates that cyt c is of great value in predicting the occurrence of CIN. Moreover, the multivariate logistic regression revealed that diabetes mellitus and serum creatinine were also risk factors of CIN. These findings are consistent with our previous research [29, 30].

There were several limitations to the present study. First, this was a single-center observational study with small sample size. The conclusions reached have certain limitations. Second, we only tested circulating cyt c levels at the time of admission. The dynamic changes in postoperative circulating cyt c levels also need to be observed. Third, we only evaluated changes in serum creatinine levels without other prognostic factors in patients with STEMI undergoing PCI. Further animal and clinical trials need to be implemented to determine the effect of cyt c on occurrence of CIN.

## 5. Conclusions

In conclusion, our study demonstrated that elevated levels of circulating cyt c are significantly associated with an increased risk of CIN. This suggests that cyt c may be a useful, reliable, and sensitive indicator for CIN after PCI in patients with STEMI.

## Figures and Tables

**Table 1:** Baseline clinical characteristics between patients with CIN and those without CIN.

Variables	CIN(n=29)	Non-CIN(n=211)	P value

Male,n (%)	18 (62.1)	161 (76.3)	0.099
Age (years)	71.07±13.77	63.06±12.7	0.002
Smoking,n (%)	14 (48.3)	118 (55.9)	0.438
Hypertention,n (%)	24 (82.8)	126 (59.7)	0.016
Diabetes mellitus,n (%)	13 (44.8)	50 (23.7)	0.015
Systolic BP (mm Hg)	127.21±22.72	128.39±22.02	0.585
Diastolic BP (mm Hg)	74.59±13.2	76.65±14.3	0.519
Heart rate (t/min)	82.59±15.99	80.33±17.4	0.486
LV (cm)	5.04±0.49	4.83±0.6	0.029
LVEF (%)	0.47±0.12	0.53±0.11	0.02
Contrast dose (mL)	109.66±23.22	105.17±15.72	0.152
Hydration,n (%)	16 (55.2)	52 (24.6)	0.001
Number of diseased vessels, n	3.1±1.59	2.78±1.26	0.336
Medication,n (%)			
Aspirin	27 (93.1)	203 (96.2)	0.433
Ticagrelor	29 (1)	210 (99.5)	0.71
*β*-Blocker	24 (82.8)	175 (82.9)	0.981
ACEI/ARB	11 (37.9)	134 (63.5)	0.008
CCB	4 (13.8)	16(7.6)	0.257
Statin	28 (96.6)	203 (96.2)	0.927
Diuretics	16 (55.2)	65 (30.8)	0.009
Hospitalization (days)	13.62±9.04	10.02±10.17	0.009
Hospitalization costs (RMB)	65245.52±30207.06	59278.88±45699.02	0.127

Data were presented as means ± standard deviation or number(%). BP, blood pressure; LV, left ventricular; LVEF, left ventricular ejection fraction; ACEI, angiotensin-converting enzyme inhibitor; ARB, angiotensin receptor blocker; CCB, calcium channel blocker.

**Table 2:** Baseline laboratory data between patients with CIN and those without CIN.

**Variables**	CIN (n=29)	Non-CIN (n=211)	P value

Troponin I (*μ*g/L)	7.25±18.29	10.13±21.22	0.957
LDL-C (mmol/L)	2.91±0.76	2.96±0.87	0.717
Uric acid (mmol/L)	346.38±82.18	332.74±118.69	0.116
Creatinine (*μ*mol/L)	119.59±44.31	94.3±48.18	<0.001
Postoperative creatinine (*μ*mol/L)	153.41±52.19	93.36±59.96	<0.001
Urea nitrogen (mmol/L)	9.37±12.2	6.19±4.25	0.004
Glucose (mmol/L)	11.36±5.98	9.11±4.61	0.035
*γ*-GT (U/L)	47.93±58.47	44.6±52.43	0.858
Albumin (g/L)	33.72±5.49	35.96±4.99	0.05
Hemoglobin (g/L)	127.83±22.91	138.6±20.57	0.012
Cyt c (ng/mL)	0.65±0.08	0.58±0.1	0.001
eGFR (mL/min/1.73m^2^)	52.08±15.63	76.27±24.15	<0.001

Data were presented as means ± standard deviation or number(%). LDL-C, low density lipoprotein cholesterol; *γ*-GT, *γ*-glutamyl transpeptidase; Cyt c, cytochrome c; eGFR, estimated glomerular filtration rate.

**Table 3:** Univariate and multivariate logistic regression analysis of CIN risk factors.

Variables	OR	Univariate analysis 95%CI	P	OR	Multivariate analysis 95%CI	P

Age	1.054	1.018-1.09	0.003	0.975	0.925-1.028	0.352
Hypertention	3.238	1.189-8.82	0.022	2.544	0.866-7.361	0.083
Diabetes mellitus	2.616	1.178-5.809	0.018	1.625	1.121-4.64	0.031
Creatinine	2.322	1.321-4.014	0.001	1.025	1.002-2.436	0.032
Uric acid	1.057	1.002-1.114	0.043	1.002	0.998-1.005	0.415
Glucose	1.077	1.009-1.149	0.025	0.962	0.882-1.05	0.385
Cyt c	13.753	5.371-28.462	0.002	7.421	6.471-20.741	0.034

Cyt c, cytochrome c.

## Data Availability

The data used to support the findings of this study are available from the corresponding author upon request.
